# Clay-Supported Metal Oxide Nanoparticles in Catalytic Advanced Oxidation Processes: A Review

**DOI:** 10.3390/nano12050825

**Published:** 2022-03-01

**Authors:** Is Fatimah, Ganjar Fadillah, Ika Yanti, Ruey-an Doong

**Affiliations:** 1 Department of Chemistry, Faculty of Mathematics and Natural Sciences, Universitas Islam Indonesia, Kampus Terpadu UII, Jl. Kaliurang Km 14, Yogyakarta 55112, Indonesia; ganjar.fadillah@uii.ac.id (G.F.); ika.yanti@uii.ac.id (I.Y.); 2 Institute of Analytical and Environmental Sciences, National Tsing Hua University, Hsinchu 30013, Taiwan

**Keywords:** advanced oxidation process, clay, metal nanoparticles, photocatalysis, nanoparticles

## Abstract

Advanced oxidation processes (AOPs) utilizing heterogeneous catalysts have attracted great attention in the last decade. The use of solid catalysts, including metal and metal oxide nanoparticle support materials, exhibited better performance compared with the use of homogeneous catalysts, which is mainly related to their stability in hostile environments and recyclability and reusability. Various solid supports have been reported to enhance the performance of metal and metal oxide catalysts for AOPs; undoubtedly, the utilization of clay as a support is the priority under consideration and has received intensive interest. This review provides up-to-date progress on the synthesis, features, and future perspectives of clay-supported metal and metal oxide for AOPs. The methods and characteristics of metal and metal oxide incorporated into the clay structure are strongly influenced by various factors in the synthesis, including the kind of clay mineral. In addition, the benefits of nanomaterials from a green chemistry perspective are key aspects for their further considerations in various applications. Special emphasis is given to the basic schemes for clay modifications and role of clay supports for the enhanced mechanism of AOPs. The scaling-up issue is suggested for being studied to further applications at industrial scale.

## 1. Introduction

Rapid developments in chemical industries enhance the quality of human life with the various products created for daily life, healthcare, building, electronics, etc. However, on the other hand, industrial activities lead to several problems related to industrial waste, including wastewater, which needs to be minimized and effectively handled without being discharged into the environment. Water scarcity problems, however, are being faced in India, China, and Thailand, as well as other countries. For example, the accessibility of safe drinking water in Indonesia is just 87.75%, and the rate can be as low as 66% in some African countries. It was reported that around 52% of rivers are heavily polluted, and India has 4% of global freshwater resources for drinking and food consumption; in addition, it was reported that about 70% of drinking water sources in China are polluted [1,2]. Moreover, water scarcity and crises are predicted to reach critical conditions as a global issue in 2025. Some studies have highlighted that trends in water scarcity are enforced by some factors, such as climate change, industrialization, food systems, country-level socio-economic systems, livelihoods and wellbeing, conflict and security, economies, and ecosystem interests [1]. Therefore, multidisciplinary approaches are required to minimize and overcome the issue. Water crises that are correlated with water contamination by hazardous substances from industrial activity need serious attention [3]. The effects of industrial waste and wastewater are mainly related to the chemicals’ components having negative effects, due to carcinogenic, infectious, and toxic properties. Chemical-containing wastewater is the main problem to be resolved for the sustainability of industries. Organic-containing wastewater from some industrial sectors represented a key interest for water quality management considerations. The potential persistence, toxicity, carcinogenic, and detrimental impacts of organic compounds utilized in many strategic sectors are the main reasons. For example, from industrial textile activities, over 20% of the dye consumed during production is released as a major component in wastewater [4]. In other sectors, pharmaceutical industries have an E-factor between 50 and 100 kg/kg of desired product correlated with many steps within the process, which are mainly organic and volatile organic compounds. The persistent character of compounds from industrial wastewater has necessitated the development of and exploration for effective and efficient treatment methods over recent decades.

Some conventional methods, such as chemical precipitation, coagulation, flocculation, adsorption, and membrane filtration, are well-known technologies which have applied for many years; however, the effectiveness is still low in terms of perspectives and challenges for sustainable technology and future perspectives [5,6]. As an illustration, coagulation and flocculation require high volumes of consumable chemicals as coagulants and flocculants which are not reusable. This contributes to the high cost of using industrial products, not only for the amounts consumed in production, but also for handling by-products and end-products [7]. Another example is adsorption mechanisms; although the adsorbents are advantageous because of their reusability, adsorbents have limited effectiveness against low concentrations of pollutants, and the recycling is dependent on adsorbent lifetimes; an adsorption capacity is also required [8]. Advanced oxidation processes (AOPs), including chemical oxidation and photocatalytic oxidation, as well as their intensified mechanisms, such as ozone-induced oxidation, ultrasound-induced oxidation, and microwave-induced oxidation, have attracted attention as powerful methods for treating organic-compound-containing wastewater [9,10]. The potential complete oxidation of organic compound pollutants in water is an improvement to reduce the chemical consumption, regeneration, and cost of the treatment. The capability of catalysts to accelerate oxidation reactions plays a key role in the effectiveness of dyes and the removal of organic compounds [11,12].

From the perspective of green chemistry and the considerable efforts for improving the catalytic process, the use of metal and metal oxide nanoparticles for advanced oxidation processes was developed. Nano-sized metal and metal oxides lead to enhanced and more efficient catalytic interactions compared with their bulk forms, and their unique characteristics make them the most versatile class of materials with properties covering all aspects of their applications [13,14]. Within this scheme, some metal oxide semiconductors were reported to have excellent catalytic activity for such oxidation process. However, some problems encountered by the use of nanoparticle forms include: (i) stability in hostile environments, especially in real and complex systems; (ii) toxicity caused by potential bioaccumulation; and (iii) regeneration and reuse technical procedures [15,16]. In vitro and in vivo studies indicate that reactive oxygen species (ROS) are produced by the exposure of nanoparticles, which is a predominant mechanism leading to toxicity. Immobilization and metal oxide nanoparticle catalyst supports are approaches for suppressing these drawbacks [17,18]. Solid supports play important roles in supporting metal oxide nanoparticles, by positioning themselves as ligands and protectors for reactive and uncontrolled conditions; these can contribute to providing an active surface site which contribute directly to the reactivity.

Silica-alumina-based materials, as well as porous materials, have been reported to be good solid supports for many metal oxide catalysts and photocatalysts [17,19]. The capability of these materials for adsorbing target compounds is a distinct advantage for efficient mechanisms which are usually studied by such kinetics and mechanism models. Undoubtedly, using clay as a natural material has always been worthy of consideration and received intensive interests. This is also specifically due to the chemical structure of clay, which can be arranged in different schemes with other materials that have fixed porous structures, such as activated carbon or zeolite. The layered structure of the clay enables the dispersion technique and mechanism to be adjusted according to the characteristics of the nanoparticles [20,21].

The use of clay minerals in catalysis itself has been recorded in oil industries since 1903. Clay structures provide surface acidity and porosity for surface mechanisms in catalytic cracking. Furthermore, many studies have recommended clay minerals as the ultimate choice for a catalyst support, due to their low cost, high mechanical and chemical stability, and environmentally friendly characteristics, including the metal and metal oxide nanoparticle catalysts [22,23,24,25].

This review is focused on clay-mineral-supported metal and metal oxide, according to the role of clay in various advanced oxidation-mechanism-based catalysis and photocatalysis processes. The study highlights the greenness of the processes, and is a fair comparison between the use of pure metal or metal nanoparticles with a clay-supported form, as well as the comparison with respect to other solid supports. Finally, the future perspectives and potential developments are presented.

## 2. Catalytic Advanced Oxidation Process for Water Treatment

**Figure 1:**
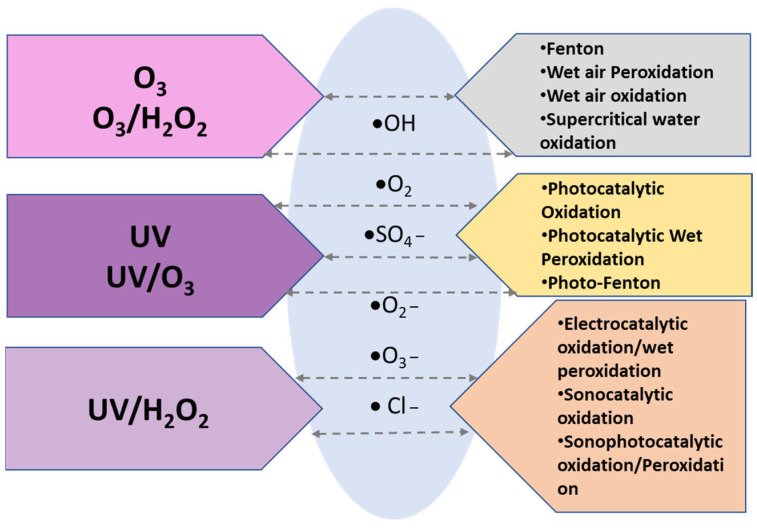
Various AOP methods.

Advanced oxidation processes (AOPs) are techniques based on the role of highly reactive species for oxidizing and degrading organic matter. The oxidation reactions are encompassed by the generation of highly reactive oxygen species (ROS), such as hydroxyl radicals (HO^●^), superoxide radicals (O_2_^●−^), and singlet oxygen (^1^O_2_). In most cases, AOPs involve the generation of reactive species other than those which have been mentioned, including ozonide anions (O_3_^•−^), hydroperoxyl (HO_2_^•^), chlorine (Cl^•^), and sulphate (SO_4_^•−^), and their radicals [26,27]. Furthermore, the mechanisms for producing those highly active ROS can be classified as chemical, electrochemical, photochemical, and sonochemical processes, as described in Figure 1.

Firstly, AOPs are known from the Fenton reaction, in which OH• and O_2_•^−^ play roles in catalysis through the presence of homogeneous Fe^2+^ in the solution. In this classic mechanism, a mixture of Fe(II) and hydrogen peroxide generates OH•, which leads to the production of HOO• and O_2_•^−^, which is known as catalytic wet peroxidation (CWP). This mechanism can be applied for wet air and supercritical systems.

**Figure 2:**
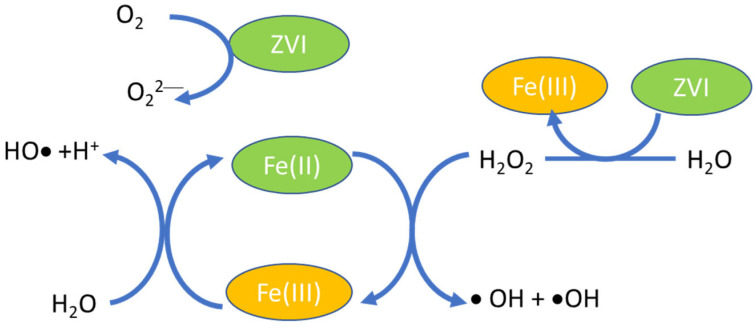
Diagram of the reduction–oxidation by Fe^2+^/Fe^3+^.

Figure 2 presents the scheme diagram of the reduction–oxidation steps:

Fe^2+^ + H_2_O_2_ → Fe^3+^ + HO• + ^−^OH

Fe^3+^ + H_2_O_2_ → Fe^2+^ + HO_2_• + H^+^

H_2_O_2_ + HO• → HO_2_• + H_2_O

HO_2_• ↔ O_2_•^−^ + H^+^

Fe^3+^ + HO_2_• → Fe^2+^ + O_2_ + H^+^

Fe^3+^ + O_2_•^−^ → Fe^2+^ + O_2_

Fe^2+^ + HO• + H^+^ → Fe^3+^ + H_2_O

For most cases of organic-polluted wastewater, advanced sulfate-radical-based oxidation processes (SR-AOPs) have received increasing attention due to their advantages, such a longer lifetime compared with hydroxyl radicals (HO•). Sulfate radicals (SO_4_•^−^) have a notably high reduction potential (2.5–3.1 V vs. NHE) and are stable at a wide range of pH values [28]. The mechanism involves peroxydisulfate (PDS, S_2_O_8_^2−^) and peroxymonosulfate anions (PMS, HSO_5_^−^) as the radical precursors for producing sulfate radicals through the presence of UV, heat, metal ions, ozone, alkaline solution, metal ions, and metal oxide semiconductors. Various transition metal ions, such as Fe(II), Fe(III), Co(II), Ru(III), Mn(II), Ni(I), Ag(I), and V(III), have been reported as effective PDS/PMS initiators, or as catalysts for catalytic oxidation [29,30]. It was noted that Co(II), Ag(I), and Ru(III) are the most effective catalysts for PDS activation; however, their high prices make them not applicable in practical water treatments. In terms of the economic considerations, Fe(II) and Fe(III) are the most commonly selected catalysts [31]. 

With the rapid advancements in catalytic techniques, there has been considerable focus on the use of various metal oxides as oxidant catalysts instead of Fe(II) and Fe(III) ions for all ROS generation mechanisms [24]. Such problems faced by the use of iron catalysts are related to corrosive properties in excessive oxygen, especially for the harsh conditions involved in wet air and supercritical processes. High pressure and temperature can easily induce corrosion problems which lead to a shorter catalyst reactor life [32,33]. On the other hand, precipitation potentially occurs, along with reduced catalyst activity. Due to severe drawbacks of the use of iron ions in homogeneous catalysis, including the lack of recoverability of the catalyst used, the replacement of homogeneous catalysts with heterogeneous forms is a growing concern. To date, the most widely applied AOPs include heterogeneous Fenton reactions, photo-Fenton reactions, catalytic wet peroxide oxidation, heterogeneous photocatalytic oxidation, catalytic ozonation, and electrochemical oxidation. Numerous other successful materials have demonstrated oxidative activity, including MnO_2_, Fe_2_O_3_, Fe_3_O_4_, TiO_2_, MnO_2_, NiO, ZnO, and SnO_2_ [5,34,35]. 

**Figure 3:**
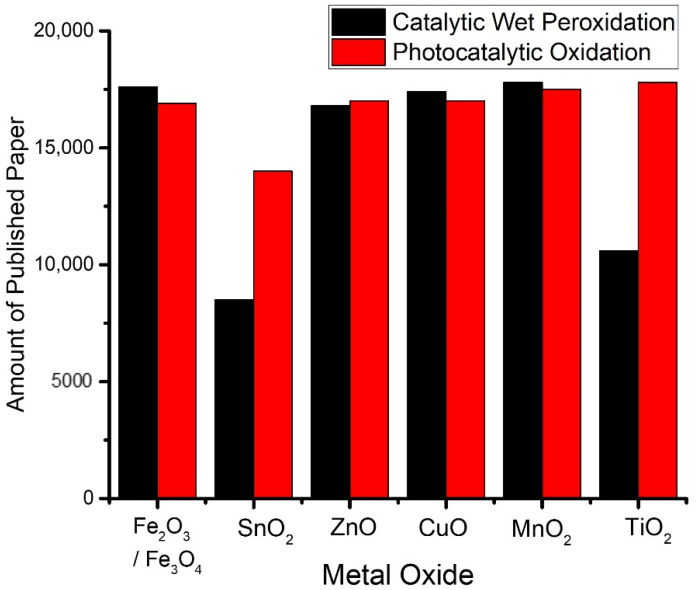
The popularity of metal oxides in AOPs observed based on publications during 2019–2020.

Figure 3 presents the popularity of metal oxides in AOPs observed based on publications during 2019–2020. 

Manganese oxide in various phases was highlighted to be a potential catalyst with a similar activity to iron-based catalysts. The possible structures of manganese oxide are α-Mn_2_O, α-MnOOH, β-MnOOH, γ-MnOOH, and Mn_3_O_4_, which influence the activity for ROS production, including PDS/PMS activation [36,37]. A notably high activity of α-MnO_2_ was recorded as the complete oxidation of phenol was attained in short-term catalytic oxidation. The enhanced reactivity of α-Mn_2_O_3_ is related to its high specific surface area and higher amounts of low-coordinated surface oxygen sites, which are capable of facilitating the activation of oxygen and improving the surface redox properties. For photocatalysis schemes, TiO_2_ is the most popular material [38,39].

## 3. Metal and Metal Oxide Nanoparticles in AOPs 

Nanomaterials and nanoparticles are at the forefront of material research and have wide applications, due to distinct properties such as better optical, magnetic, catalytic, and electronic characteristics than those of bulk or individual atoms. Metal nanoparticles exhibit larger surface-to-volume ratios as compared with their bulk equivalents, which makes them more efficient and attractive in these applications. 

Further developments were addressed to the enhanced catalytic and photocatalytic activity of those metal oxides in nanoparticle forms, including the use of some metal nanoparticles such as Ni NPs, Fe NPs, Au NPs, and their metal oxide phases [40,41,42,43,44,45]. Size effects are of great interest in many studies and conclusively indicate that the shape and size of nanoparticles are responsible for the increasing activity of reactions involving oxidation capabilities. Studies on oxidation by iron oxide nanoparticles in comparison with bulk iron oxide have revealed that different mechanisms occur on the surface. Oxidation by bulk iron oxide begins with the formation of FeO along with reduced concentrations of metallic irons; furthermore, the surface will be converted to Fe_2_O_3_ and Fe_3_O_4_ which remain as the dominant species [46]. The oxidation over iron oxide nanoparticles is noticeably different, in that the initial stage of oxidation will produce FeO on the surface which will further be oxidized almost completely to Fe_2_O_3_. These different compositions of oxides on the surface determine the more responsive surface of the nanoparticles [47]. The popularity of metal oxide nanoparticles exhibits the same trend as that presented in Figure 3, which shows that TiO_2_ NPs are the most attractive in AOPs. The applicability of TiO_2_ NPs for the photocatalytic oxidation of a wide range of organic and inorganic contaminants in water has been well-recognized. Some benefits of the use of TiO_2_ NPs are related to their band gap energy, non-toxicity, and stability. In addition to TiO_2_ NPs, many studies have also highlighted the activity of other metal oxides such as ZnO NPs, MnO_2_ NPs, and ZrO_2_ NPs for AOPs. Some representative metal oxide nanoparticles for AOPs are detailed in Table 1. 

Various methods for metal and metal oxide nanoparticles have been reported, highlighting the different forms, crystallinity, particle size, and electronic state of the nanostructures, which affects their catalytic/photocatalytic activity. Their nanostructures can be divided into zero-dimensional (0D), one-dimensional (1D), two-dimensional (2D), and three-dimensional (3D). Each of these nanostructures can be subdivided into quantum dot arrays, planar arrays, elongated arrays, and ordered structures, respectively. 

**Figure 4:**
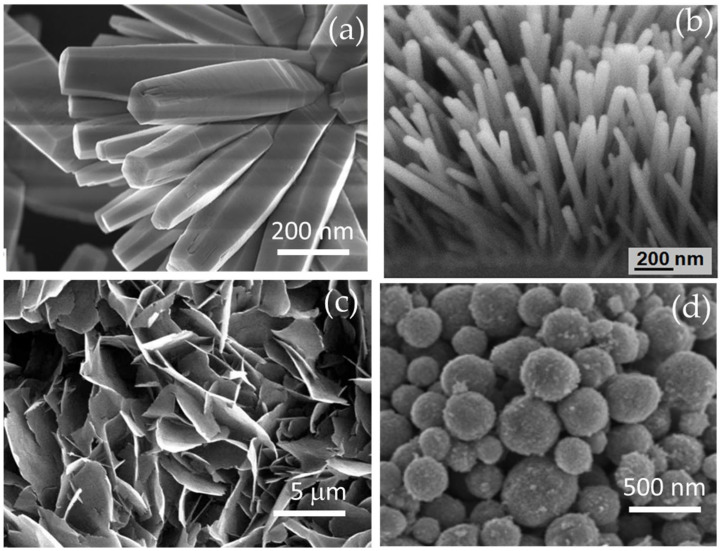
ZnO (**a**) nanoflowers, (**b**) nanorods, (**c**) nanoflakes, and (**d**) nanospheres. Reproduced from ref. [54,58,59,60] with permission from the publishers (Springer Nature, 2015; PLOS ONE, 2020; Science Publication, 2009; IOP Publishing, 2020).

As examples, Figure 4 shows the morphologies of zinc oxides with nanorod, nanoflower, and nanosphere morphologies [54,55,56,57]. 

The morphologies and particle sizes are generally governed by crystal growth kinetics and mechanisms which are directed by the synthesis method. There are various synthesis methods for metal or metal oxide nanoparticles, which are mostly dependent on the characteristics of metal or metal oxide precursor. Sol–gel, emulsion, and precipitation are synthesis mechanisms and are intensified by various techniques such as hydrothermal, solvothermal, and mechanochemical treatment. For example, in the synthesis of ZnO nanospheres, precipitation reactions can be achieved with zinc acetate and NaOH as precursors through the following reaction:

Zn(CH COO)_2_ + NaOH → Zn(OH)_2_ + CH COONa

Zn(OH)_2_ → ZnO + H_2_O

The nanospheres were obtained with different particle sizes through different techniques: hydrothermal and solvothermal [61]. For some metal oxide nanoparticles, the precursor determines the optimum synthesis method; for example, in the synthesis of TiO_2_ using titanium alkoxide, the sol–gel mechanism is the better pathway because fast gelation of the precursor can affect the aggregation and prevent the formation of nanoparticles. Other characteristics, such as corrosivity, as well as oxidizable and degradable properties of the precursor, need to be concerned. 

Further considerations are growing concerning potential environmental impacts associated with the potential toxicity towards ecosystem damage and human health. The much higher reactivity of nanoparticles compared with bulk forms. which is influenced by the particle size that facilitates their transport through cell membranes, is a characteristic which needs risk assessment. [62,63]. Nanoparticle immobilization into stable and supportive solids is one of the strategies for preventing their release into the environment; moreover, metal and metal oxide incorporation with inorganic solids to form nanocomposites can enhance the reusability and recyclability of the catalyst without losing activity. Some research has evaluated the increased catalytic efficiency related to the cost-effectiveness and catalyst capabilities. For example, the dispersion of Pt nanoparticles into mesoporous Co_3_O_4_ was reported to increase the catalytic oxidative degradation efficiency toward methylene blue (MB) dye, with almost 100% removal after 60 min of treatment [64,65]. Similar results were recorded from supported ZnO nanoparticles using zeolite [66,67], supported iron nanoparticles using zeolite [68,69], and supported Au nanoparticles into TiO_2_ and BiVO_4_ [70,71]. Microporous and mesoporous solids were employed for supporting metal and metal oxide nanoparticles; some of them are noted to be low-cost supports such as zeolite, silica, graphene, carbon, C_3_N_4_, and clay minerals [72,73,74,75,76]. Especially for photocatalytic mechanisms, the enhanced activity and stability of metal or metal oxide nanoparticles can be attributed to the charge separation mechanisms provided by the solid support. Improvements in the sorption capacity are common benefits from the porous structure of the support. In addition, the capability of the surface to adopt the metal ions is notable. The presence of functional groups associated with hydroxyls bound on carbon material is an example which can be achieved by the utilization of biomass-based support [75]. In γ-Fe_2_O_3_/graphene oxide (GO), the enhanced photo-Fenton activity, as identified by the accelerated Fenton-like mechanism, is caused by charge separation between g-Fe_2_O_3_ and GO [75]. 

Supports with a high specific surface area tend to support catalysis via an adsorption mechanism. Activated carbon is one of the verified supports for this model, because the supporting Au on activated carbon exhibits a higher turnover frequency (TOF) in catalytic phenol wet peroxidation and requires less H_2_O_2_ as an oxidant [77]. A similar role of the support was also demonstrated by clay in Fe/Cu/Zr-pillared clays for the catalytic wet peroxide oxidation (CWPO) of 4-nitrophenol (4-NP). The higher TOF was achieved with a smaller amount on the active catalytic site [78]. Interestingly, the synergic effect between active nanoparticles and the support leads to better reusability and recyclability [79,80,81]. 

## 4. Clay Structure and Utilization in Catalysis 

Clays are silica alumina minerals with layered structures composed of aluminum silicates which are formed by tetrahedral and octahedral sheets, where the layers possess net negative charges and contain cations, such as Na^+^, K^+^, Ca^2+^, etc., which occupy the interlamellar space. The amenability of clays for modification lies in the exchangeable properties of the cations; as such, they can easily be replaced by other cations or other molecules. The ion exchange mechanism and the layered structure govern the possible insertion of metal cations and molecules to be covalently anchored to layer atoms. The cation exchange capacity of clay is one of the factors affecting the modification mechanism, and is directly related to the clay structure, which depends on its chemical composition. In this regard, clay minerals are classified into different groups as follows: kaolinite, smectite, vermiculite, illite, and chlorite [82]. 

The smectite class of clay is the most popular for supporting metal and metal oxide catalysts, not only for advanced oxidation processes, but also for some catalyzed organic reactions. Smectite is composed of two layers of tetrahedral silica and one layer of octahedral alumina group, alternatively known as a 2:1 layer or T-O-T clay minerals, belonging to a group of hydroxyl alumino-silicates [83]. Smectites, which include montmorillonite, saponite beidellite, nantronite, and hectorite, are naturally formed from the weathering of soils or volcanic ash. The smectite group of clays is distinguished by differences in the chemical composition, with substitutions of Al^3+^ or Fe^3+^ for Si^4+^ in the tetrahedral cation sites and Fe^2+^, Mg^2+^, or Mn^2+^ for Al^3+^ in the octahedral cation sites [84]. 

Smectite structures increase swelling capabilities due to the high cation exchange capacity and ease to be hydrated. The thin layers and small particle sizes contribute to their high surface area; hence, they exhibit a high degree of absorbency of many materials, including organic compounds. Similarly, the structure is consistent for vermiculite, which is also 2:1 or T-O-T clay. Vermiculite differs from smectite due to its isomorphous substitution of higher-valence ions by lower-valence ions in the tetrahedral sheet (e.g., Al^3+^ for Si^4+^) and in the octahedral sheet (e.g., Mg^2+^ or Fe^2+^ for Al^3+^); thus, it has more negatively charged plate surfaces [85,86]. 

Different from smectite and vermiculite, kaolinite is a 1:1-type clay mineral which is composed of one layer of tetrahedral silica and one layer of an octahedral alumina group (T-O). Some minerals include kaolinite, nacrite, dickite, and halloysite, with a chemical formula of Al_2_O_3_·2SiO_2_·2H_2_O (39% Al_2_O_3_, 46.5% SiO_2_, and 14.0% H_2_O) [87,88,89]. The structure possesses strong binding forces between the layers, causing less hydration or expansion resistance when wetted, a low specific surface area, and cation exchange capacity. Chlorite has a 2:1:1 structure with a sequence of T-O-T-O, and crystalline interlayer characteristics. Even though there is no possibility for intercalation, kaolinite and chlorite minerals have been reported to exert a capability to adsorb organic molecules and support metal and metal oxide nanoparticles.

Referring to the availability of pore structure, Brønsted and Lewis acid sites in clay structure, clays have been utilized in the beginning of petroleum refining and petrochemical industries. Kaolinite was the main clay mineral which was utilized in petroleum refining as active cracking catalyst [90]. Referring to the principle of heterogeneous catalysis, the availability of porous structure in clay minerals can accelerate the reaction via surface mechanism. The cracking mechanism occurs and is controllable when the feed in gas phase is adsorbed on the catalyst surface, for furthermore breaking down into smaller molecules. The presence of Brønsted acid on the surface accelerates the cracking mechanism. 

However, the lower stability of raw clay was found due to the loss of the surface area at high temperature-forced advanced modifications. Acid treatment and cation exchange processes were applied to clay in order to increase Brønsted or Lewis acidity, a procedure that was patented in 1949. In addition, for acid-catalyzed organic reaction, the selectivity to produce a certain product was the important feature to be concerned as well as total conversion of the reaction. The high specific surface area (SSA) and structure of clay minerals bring about the potencies to be modified and act as effective supports rather than its individual use. The benefit of previous reviews allows us to focus on material most relevant to modern, more relevant green/clean technologies. Metal modifications to clay structure, such as Pt-, Ni-, and Co- supported clay, were established as active catalysts in hydrocracking and hydroprocessing of fuel. Furthermore, clay modifications were performed in many schemes which were adapted to the reaction that will be catalyzed [91]. Functionalizations with basic modifier were attempted for base-catalyzed reactions, such as transesterification reaction, to produce biodiesel, for example, by supporting CaO and CaO/KF [92,93]. For other organic reactions with specificity and selectivity to produce a certain isomer, the inorganic complex immobilization was also reported, for example, Schiff base Pt(II)-complex intercalated montmorillonite. The catalyst showed 100% selectivity in producing 2,4-dinitrotoluene from the reduction of nitrobenzene [94]. The immobilization of metal complexes into clay structure, mainly the swelling clay, gave several advantageous related to the reusability, stereoselectivity, and minimizing radical mechanism that reduce applicability in producing selective product. Other examples for this are chiral bis(oxazaline)-copper (Box–Cu(II)) complexes supported on laponite clay for the addition of alkoxycarbonyl carbene-reaction [95], and ruthenium-complex intercalated saponite for citral conversion [96]. Until now, the study on clay functionalizations for organic reactions catalysis is still developing with various mechanisms with respect to the green chemistry principles consideration, including the metal and metal oxide nanoparticles modification which are reviewed in this paper.

## 5. Clay Modifications 

There are some modification procedures to functionalize the clay surface. Below are some important schemes for this. 

### 5.1. Impregnation 

The dispersion of metal and metal oxide nanoparticles into non-swelling clays, such as kaolinite, halloysite, dickite, and nacrite, is usually conducted by the impregnation method, even though the procedure can also be conducted for swelling clay. 

The impregnation mechanism depends on the metal precursor, and can be assisted by severe intensification procedures such as hydrothermal, microwave, and ultrasound irradiation. Various techniques, such as the sol–gel method, coprecipitation, wet impregnation, and their combinations, have been performed and chosen based on the technical characteristics of the metal precursor. 

Sol–gel dispersion is usually performed because metal oxide formation can be achieved through polymerization in sol–gel systems. Pillarization by using organometal precursors, such as metal alkoxides (such as titanium isopropoxide, titanium isobutanate, and zirconium isopropoxide), and acetate salts, usually occur within this mechanism. Wet impregnation and coprecipitation are based on the homogeneously dispersed metal salt in the clay suspension, in which the coprecipitation conducted by the additive base environment forms the deposited metal hydroxide. 

The dispersion of TiO_2_ in kaolinite and hectorite using titanium isopropoxide can be conducted by a sol–gel mechanism due to Ti-O- bond formation via polymerization [88]; using different precursors, such as TiOCl_2_ and TiOSO_4_, the impregnation can be conducted by depositing titanium salt into clay suspension or through the coprecipitation method. Temperature, pH, and the precursor solvent are crucial factors in determining the particle size and distribution of the metal oxide in clay supports. Research on TiO_2_/kaolinite using titanium isopropoxide demonstrated the role of the DMSO solvent in producing more space for the more homogeneous dispersion of TiO_2_, which furthermore determines the hydrophobicity and reactivity of the nanocomposite [97]. The impregnation method including wet impregnation consists of mixing a precursor salt with a clay suspension in a solvent and tuning the optimum pH. As an example, the impregnation of TiO_2_ into kaolinite utilizing TiOSO_4_ was optimally conducted in an acidic mixture in order to maintain the ionic form of Ti^4+^ in the solution [98]. 

The coprecipitation method was reported in the synthesis of ZnO/montmorillonite. The combination of the sol–gel mechanism and wet impregnation method can also be conducted. 

In a different scheme, the composite formation of MnO_2_ nanosheets and MnO_2_ nanowire with montmorillonite was achieved under hydrothermal conditions. For both inserted MnO_2_ nanostructures, KMnO_4_ solution was employed as a precursor and a hydrothermal procedure was conducted in a Teflon-lined stainless-steel autoclave. MnO_2_ nanosheets were formed at 160 °C for 24 h over the mixture of the precursor solution and montmorillonite suspension. Meanwhile, for MnO_2_ nanowire, a combination of KMnO_4_ and (NH_4_)_2_S_2_O_8_ under hydrothermal treatment at 90 °C for 12 h was performed. 

**Figure 5:**
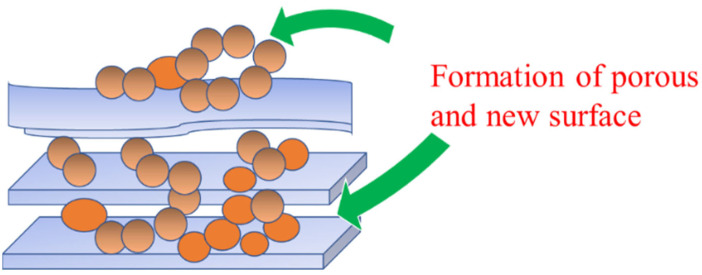
The possible formation of porous structures and new surfaces as adsorption sites by metal/metal oxide impregnation.

A decreased specific surface area was observed from both the nanostructure insertion to the montmorillonite structure, although a fast and efficient catalytic oxidation was reported. The catalytic activity is also subjected to the enhanced adsorption capacity caused by the surface oxide structure of the nanostructures [99]. The impregnation method does not guarantee a reduced specific surface area of the nanomaterial; however, in some cases, because the dispersed metal oxide is in the nanoparticle form, the increasing specific surface area related to the homogeneous surface dispersion results in more space formed on the surface. An increased specific surface area, about twice as large as that compared with the raw material, was recorded from the Fe_2_O_3_ nanoparticles deposited on kaolinite [100]. Figure 5 illustrates the possible formation of porous structure and new surfaces as adsorption sites by metal/metal oxide impregnation. 

### 5.2. Pillarization 

**Figure 6:**
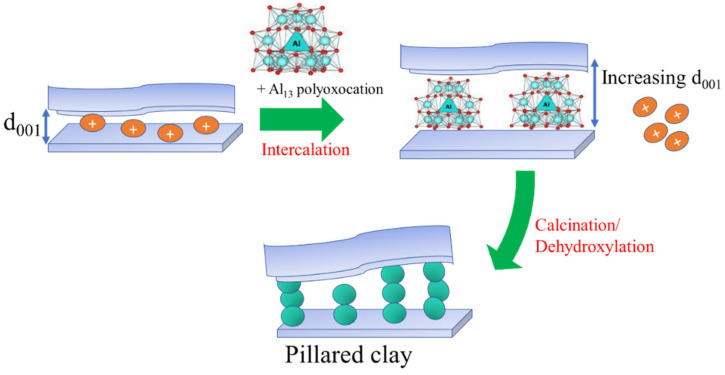
Schematic representation of clay pillarization.

Pillarization is a popular mechanism of clay modification by metal oxide insertion to swelling clays such as smectite and vermiculite. The process consists of intercalation followed by a calcination procedure. Intercalation is an ion exchange process of native cations between the smectite layers with other cations having a higher reduction potential, or the polyoxocations [78,101]. The intercalation is intended to open up the interlayer region, creating a higher specific surface area or pore volume; furthermore, through the calcination process, dihydroxylation of the polyoxocations will produce a metal oxide as metal oxide pillars which is also homogeneously distributed metal oxide (Figure 6). The stability of polyoxocations is the main factor for pillarization; therefore, the optimum conditions for some metal oxide pillaring precursors were studied. The polyoxocation of the Keggin ion Al_13_ is reported to be the best pillaring precursor for Al_2_O_3_, which can be prepared by the slow titration of Al salt with ^−^OH at an Al:OH molar ratio of 1:1. With the same metal:OH molar ratio, the pillaring precursor for SnO_2_ pillarization is synthesized by the mixing or slow titration of Sn salt with ^−^OH with a final pH of 2, and Zr salt with ^−^OH at a pH of 6–7.

The trimer Sn_3_(OH)_4_^2+^ is the predominant species from the hydrolysis to construct stable SnO_2_ in pillar formation [102]; meanwhile, the pillaring precursor for ZrO_2_ is in the tetramer form (Zr_4_(OH)_14_(H_2_O)_10_)^+^ [103,104,105]. Different authors have demonstrated that the preparation of Fe-PILCs is affected by the OH:Fe molar ratio [105,106,107,108,109]. The polyoxocation of iron pillaring solution was obtained to have optimum polymerization at a pH of between 1 and 1.8; in addition, pH > 1.8 caused precipitation [110,111]. 

Pillaring species for titanium-pillared clay are still not exactly established. Some papers have conclusively suggested that in acidic environments, a polymeric [(TiO)_8_(OH)_12_]^4+^ complex is the polyoxocation, and can be achieved with the utilization of TiCl_4_. Another species is trimeric titanium hydroxide [85]. Various precursors and synthesis routes can be performed for the synthesis of titanium-pillared clay (Ti-PILC). TiCl_4_, TiCl_3_, TiOCl_2_, titanium tetraisopropoxide, and tetrabutyl titanate have been reported as titania precursors [112,113]. The use of titanium tetraisopropoxide and tetrabutyl titanate was reported to be better in terms of specific surface area and clay structure compared with TiCl_4_ and TiOCl_2_. The milder condition achieved refers to the stability of TiCl_4_ and TiOCl_2_ at a very low pH, which potentially destroys the structure of clay minerals [85,114]. Conversely, the large Ti uptake in Ti-pillared clay was observed when using TiCl_4_ as the intercalating agent; up to 55% as compared with the use of Ti(EtO)_4_ and Ti(isop)_4_ as precursors, which gave uptake values of TiO_2_ of ~30%. As organo-titanium compounds, the interaction between precursors and native cations was not in ion exchange mode; thus, the release of exchangeable Ca^2+^ and Na^+^ cations did not occur. In contrast, this occurred in the use of TiCl_4_ or TiOCl_3_ [115]. 

The calcination process or sintering temperature of polyoxocation-intercalated clay is a crucial factor, mainly for the formation of the metal oxide nanoparticles phase. The formation of the metal oxide phase during the sintering process is thermodynamically controlled by thermal change, including the set temperature and heating rate. Along with the increasing particle size, clay destruction may occur due to the rapid heating rate, which is directly expressed by the reduced specific surface area. The pillaring of hectorite and montmorillonite with ZnO demonstrated these variable synthesis outcomes [116,117]. Comparing the formation of metal oxide phase in pillared clay form with the formation of pure metal oxide nanoparticles is a better approach. As an example, the formation of ZnO nanoparticles is governed by the sintering temperature. As a photocatalyst, the wurtzite phase can be achieved at a temperature of 700 °C, which also affects the morphology of the nanoparticles [116]. Particularly for TiO_2_, the increasing temperature tends to produce a rutile phase instead of an anatase phase, which expresses less band gap energy. This considerably influences the optical properties of the material [118]. As an example, the varied temperature from 300 to 800 °C, and revealed the optimum anatase:rutile ratio at a temperature of 500 °C. 

For specific applications, the combination of two metal oxide pillars was also synthesized. For example, in order to provide photoactivity with a high specific surface area of the nanocomposite, a combination of Al_2_O_3_ and Fe_2_O_3_ was established. The aluminum pillarization of clay is well-known to result in an increased specific surface area due to the stability of polyoxocation of the Al_13_ Keggin ion; moreover, the photocatalytically active Fe_2_O_3_ in a homogeneously distributed form contributes as a photoactive material. Similarly, combinations of Cu/Al-, Zn/Al-, and Cr/Al-pillared clays have been reported. Cu/Al-pillared clay was prepared as a combination of intercalated poly(hydroxy)aluminum (Al_3_(OH)_4_^5+^) and copper Cu_3_(OH)_4_^2+^ species, using slow titration to produce AlCl_3_ and CuCl_2_. The molar ratio of Cu^2+^:(Al^3+^+Cu^2+^) in the precursor solution affected the increasing d_001_ and specific surface area [119]; the optimum synthesis conditions were a Cu:(Al+Cu) molar ratio of 0.1:1 and OH:Al = 2.5:1 [120]. Those ratios determined the characteristics of the material, which play an important role in the electrocatalytic degradation of CO_2_ as well as oxidative features in CWPO. 

Studies on the combination of the mixed metal pillarization of Al/Fe-pillared clays revealed the specific contributing properties of each metal for such catalytic activity. Based on a solid state study using ESR and 27Al-NMR spectroscopy, mixed pillars exhibiting a Keggin-like formula [FeAl_12_O_4_(OH)_24_(H_2_O)_12_]^7+^ (FeAl_12_^7+^) were utilized as active species in the pillaring process [121]. Table 2 provides some crucial factors for the synthesis of pillared clay. 

**Figure 7:**
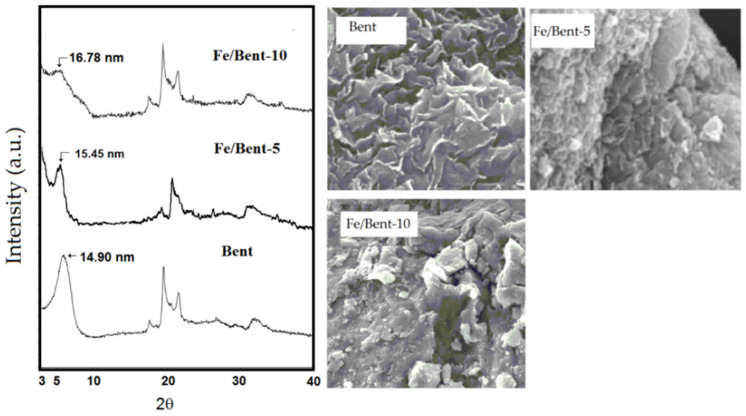
XRD patterns and SEM images of Fe-pillared bentonite with varied Fe content (5 and 10 mmol/10 g). Adapted from Ref. [123] with permission from BCREC Group.

**Figure 8:**
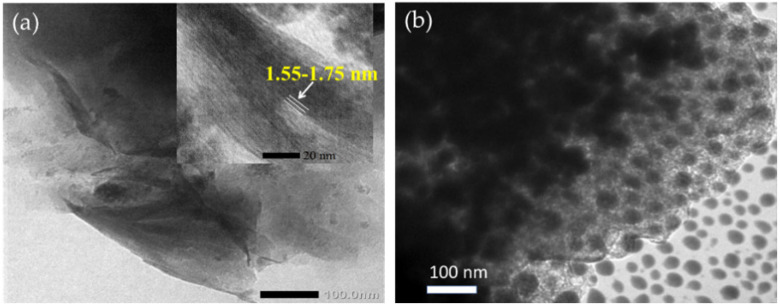
TEM images of SnO_2_/montmorillonite at Sn/montmorillonite ratios of (**a**) 2.5 and (**b**) 10.0 mmol/10 g. Adapted from Ref. [80] with permission from the Elsevier B.V., 2021.

From the schematic representation of clay pillarization, the increasing basal spacing, d_001_, is an important earmark for the success of pillarization, which influences the surface morphology. Figure 7 shows the increasing d_001_ features from Fe_2_O_3_-pillarization to bentonite identified by XRD and SEM analyses [123]. The shift in d_001_ reflection to lower angle indicated the enhanced interlayer space, from 14.9 nm in the bentonite sample (Bent) to 15.45 nm and 16.78 nm for Fe_2_O_3_-pillared bentonites with Fe contents of 5 mmol/10 g and 10 mmol/10 g (Fe/Bent-5 and Fe/Bent-10), respectively. This is also reflected by the evolution of the surface morphology of the sample to be flakier in structure, as indicated by the immobilized Fe_2_O_3_. Similar patterns of XRD and SEM results were reported for TiO_2_, ZnO, and other metal oxides [113,137]. The change in interlayer space could also be confirmed by TEM analysis, in which an example from SnO_2_ pillarization is presented in Figure 8. In addition, at the high concentration of metal oxide, such dispersed nanoparticles are identified beside the fringes correlated with the d_001_ of the clay structure (Figure 8a,b) [138,139]. 

When using natural clay, the mineral composition strongly influences the characteristics of the pillared clay. A composition of smectite and illite, and the presence of a non-swellable mineral, such as quartz in the material, has the effect of decreasing the capability of clay to be intercalated with the polyoxocation of the precursor [127]. Pretreatments, including the demineralization of impurities through the addition of hydrogen peroxide, as well as refluxing the minerals in acid, were reported to be effective to increase the basal spacing, d_001_, of clay, along with the increased specific surface area [140,141,142]. Intensive studies on the effects of initial clay were also carried out for the synthesis of porous clay heterostructure [143]. 

**Figure 9:**
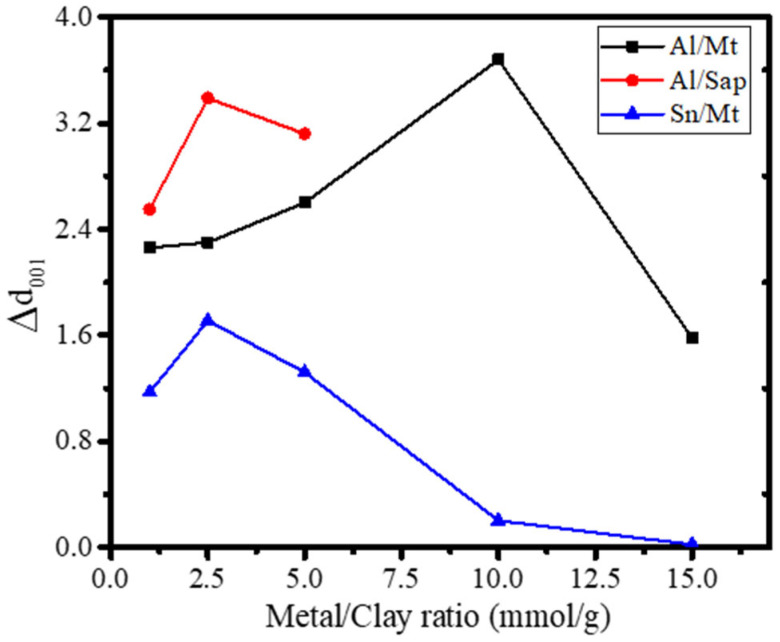
The effect of the metal:clay ratio on the Δd_001_ of pillared clays.

The metal-to-clay ratio determines much of the catalytic and photocatalytic activity, governing the physicochemical character of pillared clay. From many studies, such as research on the synthesis of TiO_2_-pillared montmorillonite and SnO_2_-pillared montmorillonite, it can be concluded that the metal-to-clay ratio is not linearly correlated with increasing either the physical characteristics or the catalytic performance of the pillared clay, but it has an optimum condition, which depends on the electronic properties of the surface, the cation exchange capacity of clay, and the affinity of metal polyoxocations to enter the intercalating region [80,144]. Figure 9 presents the effect of the metal:clay ratio on the d_001_ of pillared clays, indicating the optimum conditions.

Figure 9 demonstrates that the metal:clay ratio for different metals and clay minerals has an optimum condition to increase d_001_, as identified for the created porous structure and facilitating the specific surface area. 

The change in clay structure by metal oxide pillarization is not only related to surface parameters including the specific surface area and morphology, but is also reflected by the change in the electronic properties of the metal oxide. The higher band gap energy of TiO_2_ in the pillared saponite (3.25 eV) compared with the band gap energy of TiO_2_ (3.20 eV) was found to correspond to the smaller TiO_2_ particle size in the nanocomposite form [145,146]. This is characteristic for the homogeneous distribution of the metal oxide. In addition, the opposite phenomenon of the lower band gap energy occurs, as related to the possible crystal defects. This trend was demonstrated by SnO_2_ pillarization into montmorillonite. The band gap energies of SnO_2_-pillared montmorillonite are within the range of 2.49–3.18 eV, depending on the Sn content, and are lower than that of bulk SnO_2_ (3.6 eV). Oxide formation in the interlayer region is not as simple as that in the bulk form; therefore, the defects in the crystalline system cause changes in the electronic structure of SnO_2_ [80].

**Figure 10:**
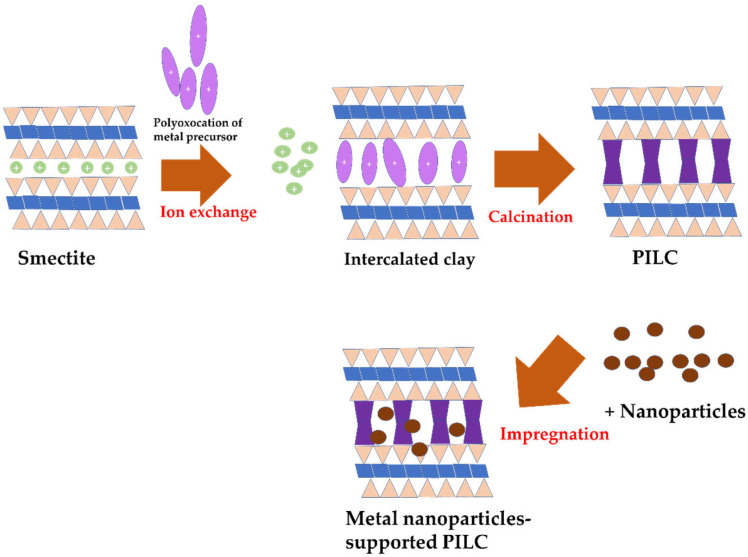
Schematic representation of metal nanoparticle impregnation onto PILC.

In addition to clay pillarization with mixed metals, combined metal/metal oxide dispersion into the clay structure can also be conducted, utilizing single-metal-oxide-pillared clay as a support for another metal or metal oxide nanoparticles. This scheme takes two steps: pillarization, followed by an impregnation procedure, as described in Figure 10. 

Combinations such as TiO_2_-impregnated Fe-pillared clay (TiO_2_/Fe-PILC), Fe-impregnated Co-pillared clay (Fe/Co-PILC), Ru-impregnated Ti-pillared clay (Zn/Fe-PILC), and Co/Al-PILC exhibited hybrid properties for gaining either homogeneous dispersion or material stability, which are sufficiently accommodating for surface interactions for the catalytic process [147,148,149,150]. The immobilized Co^2+^ and Sn^2+^ have been proven as successful catalysts for dye oxidation. On tartrazine oxidation using oxone, Co/Al-PILC was demonstrated to be a stable and efficient catalyst in the degradation of both tartrazine and the detected products [150].

The homogeneous expansion of the clay layers depends on the distribution of polyoxocations during the intercalation step, and dihydroxylation during calcination. Studies of the synthesis of ZrO_2_/bentonite demonstrated that the homogeneous clay layers were a function of the diffusion of ions in the interlamellar space. The most effective polyoxocation in the synthesis was in the form of hydroxycation [Zr_4_OH_8_ (H_2_O)_16_]^8+^, which rapidly polymerizes by the mixture of ZrCl_4_ and NaOH at an OH:Zr ratio of 4:1. The order of polymer species which determines the intercalating layers changed as the ageing temperatures changed. From varied ageing temperatures between 25 and 100 °C, it was found that different species sizes caused the irregular stacking of clay layers, and the optimum temperature was 40 °C. The amorphous condition was obtained at 100 °C [136]. Furthermore, the calcination temperature is a meaningful variable for determining not only the homogeneity of metal oxide dispersion, but also the metal oxide phase [124]. In the synthesis of pillared clays, the distance between the sheets of clay structure results from the dehydration and dehydroxylation of intercalated metal polyoxocations along with the formation of metal oxide as permanently linked adjacent layers. Therefore, the optimization of dihydroxylation temperature for such intercalated cations is a crucial step. 

### 5.3. Porous Clay Heterostructure

**Figure 11:**
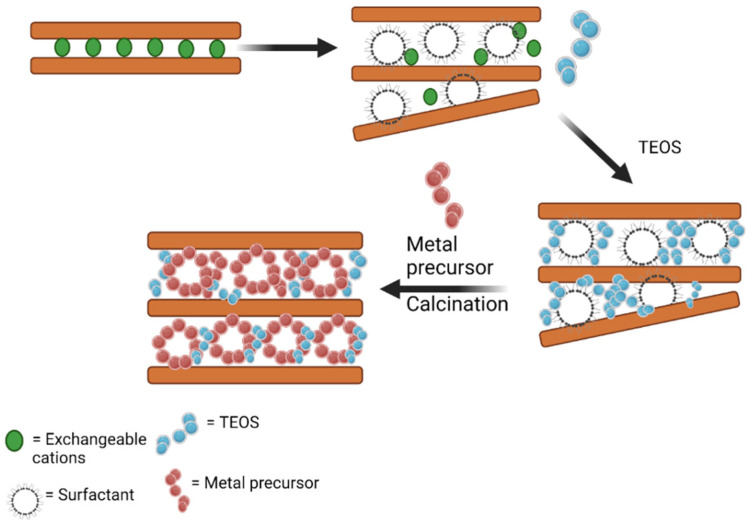
Schematic representation of porous clay heterostructure synthesis.

The creation of a mesoporous structure can also proceed through the combination of cationic precursors incorporated with silica or titania pillars, known as a porous clay heterostructure (PCH). The higher interlayer distance of the basal spacing of clay structure within the range of 3.02–3.49 nm is obtained with templating agents, such as cetyl trimethyl ammonium or other organic compounds, to form a bigger space for positioning the metal oxide arrangement, as can be observed in Figure 11. From several studies, it was found that the deposition of metal oxide within the PCH structure produced well-dispersed forms [151]. The increasing basal spacing, d_001_, was within the range of 0.1–3.0 nm, and could be obtained by clay pillarization which depends on some crucial factors in the synthesis such as the stability of polyoxocations, pH of the intercalation, calcination temperature, and metal content. Surface modification using a surfactant prior to polyoxocation intercalation facilitates the assembly of nanoparticle formations of the metal via controlling agglomeration and directing the homogeneous dispersion of the metal. The larger pore opening related to the molecular size of the organic compound is a crucial factor for the creation of porous structures [131,134]. The organic matter from the surfactant was removed by thermal treatment at 500 °C. A significantly increasing specific surface area was also reported by dispersed ZnO in the PCH formation from saponite, in comparison with the ZnO/pillared saponite [152]. With the same amount of Zn (10 mmol/g), ZnO-pillared saponite exhibited a specific surface area of 188 m^2^/g; meanwhile, ZnO/PCH showed 770 m^2^/g. Similar phenomena were also reported in the PCH synthesis of ZnO-TiO_2_/delaminated montmorillonite in comparison with the pillared form [153]. The increasing porosity brings increased thermal stability for some PCHs. For example, in the synthesis of the porous structure of titania/silica-montmorillonite [151], it was found that the silica–titania clusters were formed by separated tetracoordinated titanium cations incorporated into the silica pillars. The cluster formation was found to be enhanced, demonstrating thermal stability at temperatures exceeding 600 °C.

It can be seen from Table 2 that the physicochemical characteristics of pillared clays are mainly governed by either the molar ratio of metal-to-base, or the metal-to-total-metal in the pillaring precursor, in addition to the ratio of metal-to-clay-mass [80]. Studies on the effect of the Sn-to-montmorillonite mass ratio described the presence of an optimum point for increasing the basal spacing and specific surface area of montmorillonite. 

### 5.4. Clay Modification with Metal Nanoparticles 

The dispersion of metal nanoparticles into the clay structure can be conducted by either an ion exchange process of the metal ions with native cations of the clay mineral, or the in situ dispersion of metal nanoparticles into the clay structure. The dispersion of nanosized zero valent irons (ZVIs) into montmorillonite structure by the ion exchange method was conducted, resulting in homogeneously distributed nanoparticles [154]. After the ion exchange process had reached surface equilibrium, the mild reduction of inserted Fe^2+^ was achieved with NaBH_4_. Similarly, the dispersion of Cu nanoparticles into montmorillonite was conducted by exchanging cations with CuCl_2_·2H_2_O with consideration of the cation exchange capacity, followed by reducing exchanged Cu^2+^ with hydrazine to form Cu nanoparticles (Cu NPs) [155]. The same scheme was also performed for the dispersion of Ni NPs in montmorillonite [156] and Pd NPs [157]. The in situ metal nanoparticle dispersion was actually similar to the ion-exchange method, but the certain amount of metal was chosen without consideration of cationic equilibrium [158,159,160]. The dispersion of Au NPs into attapulgite is an example where Au NPs are prepared by the most commonly published method—Frens. The reduction of Au from a HAuCl_2_ precursor with citric acid was conducted prior to dispersion with attapulgite [161] and montmorillonite [162]. The ultrasound-assisted dispersion of colloidal Au NPs into montmorillonite was proposed as a technique to yield homogeneous dispersion [163]. 

**Figure 12:**
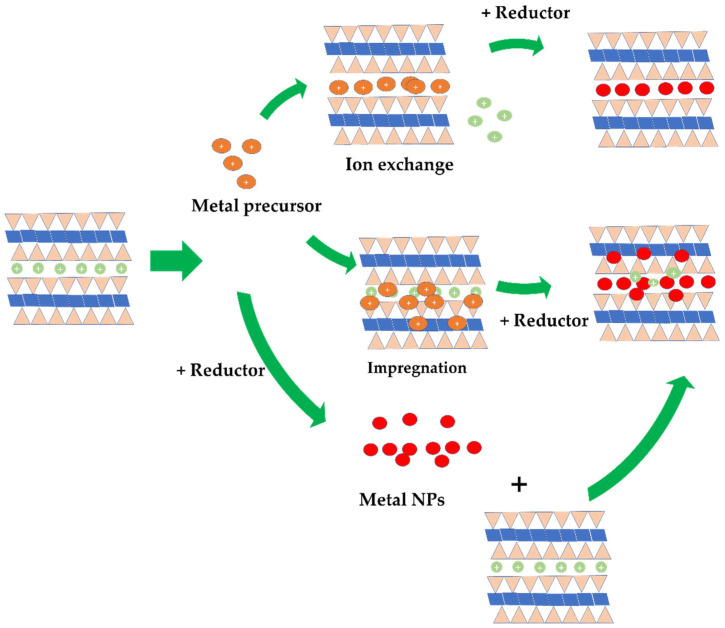
Schematic representation of the dispersion of metal nanoparticles in clay structure.

The impregnation of green synthesized nanoparticles is a novel method reported by several recent papers. Metal nanoparticles are prepared by mixing precursor salt with a plant extract at a certain volume ratio and reaction condition to produce the nanoparticle solution. The solution is then dispersed into clay suspension to produce the nanocomposite. The greenness of using plant extract as a bio-reductor as a replacement for common reductors such as NaBH_4_ and other corrosive chemicals is the main consideration for the popularity of this method [158,164]. In addition to the reductor for NPs formation, the secondary metabolites from the plant extracts play roles in maintaining the stability of the nanoparticles. Some examples from this scheme are kaolinite-supported bio-fabricated Ag NPs using *Murraya koenigii* fruit extract [165], montmorillonite-supported *Urtica dioica* leaf-extract-mediated Ag NPs [165], and montmorillonite-supported Ag NPs fabricated using *Ocimum basilicum* L. and *Teucrium Polium* L. [166]. Montmorillonite-supported Ni NPs were also prepared through a similar scheme [167]. Figure 12 is a schematic representation of the synthesis method. 

In addition to their excellent activity as antibacterial agents, clay-supported Ag NPs were established as versatile catalysts for the degradation of nitroaryl (4-nitrophenol) and methylene-blue-containing solution [168,169]. Ni NPs/montmorillonite presented excellent catalytic activity in the oxidation of methanol [167]. 

The applicability of modified clay for industrial applications is a crucial issue. The scaling-up process of modified clay preparation faces challenges mainly related to intercalation, washing, and drying steps [56,170]. Minimizing water use in the synthesis was attempted through utilizing high concentrations of clay suspension in the intercalating metal oxide precursor and optimizing water for neutralization/washing [171]. A successful scaling-up study was reported for Al/Fe-pillared clay synthesis by a factor of 1000, from the lab (10 g) to the pilot scale (10 kg) [172]. Referring to the complexity of attaining optimum conditions in other metal oxide pillared clays, optimization for scaling-up is an important aspect for development. 

### 5.5. Intensification on Metal/Metal Oxide-Supported Clay Nanocomposite

Referring to the green chemistry principles and applicability for industrial scaling-up, minimizing energy and time required for the synthesis of clay-based nanocomposites is under consideration. In this regard, intensification procedures for the intercalation step have been widely reported. Several techniques are detailed subsequently. 

#### 5.5.1. Mechanochemical

The mechanochemical synthesis of nanocomposites involves a high-energy milling technique and is usually carried out at controlled pressure. The intercalation of metal/metal oxide into the clay structure can be achieved with this method. For example, Li et al. (2020) synthesized a ternary polyethyleneimine clay nanocomposites using a mechanochemical technique for the removal of heavy metal pollutants in wastewater [173]. Mechanochemical techniques can produce different physicochemical properties of materials, such as changing the particle/crystal size, changing the lattice structure, changing the morphology, increasing the surface area, and even generating structure defects. Therefore, this technique is quite effective for the intercalation process on clay structures, because physically, the clay structure will undergo a rearrangement that enables easy modification and intercalation. However, this method also has several disadvantages: (1) the small nanoparticles formed have low homogeneity, meaning that the particle size cannot be appropriately controlled; and (2) contamination from the grinding material causes a lower purity of the nanocomposite material. 

This mechanochemical method has been widely used to synthesize metal/metal oxide intercalated in clay. In this method, nanocomposites can be prepared by direct mixing of the constituent materials. Here, several important factors that determine the characteristics of the resulting nanocomposite are the purity of the raw material, the type of raw material, the distribution and size of the resulting particles, and the degree of agglomeration in the process [174]. Contamination of the grinding medium can be avoided by using appropriate materials such as zirconia, alumina, silicon, and tungsten carbide. Yang et al. (2021) prepared zero-valent aluminum (ZVAl)-clay nanocomposites using ball milling with agate balls [175]. The modification of ZVAl-clay showed excellent degradation for 4-chlorophenol with an improved efficiency of around 38.7%. This method is more suitable for synthesizing composites derived from raw materials such as powders. Although this method is highly suitable for the preparation of various nanocomposites and in intercalation processes, the main drawback of this method is the low particle size homogeneity. 

#### 5.5.2. Microwave Irradiation

The synthesis of metal/metal-oxide-pillared clay nanocomposites has been widely reported and optimized for producing excellent chemical activity properties. Foroughi et al. (2017) synthesized CdO/clay nanocomposites via simple microwave irradiation [176]. Their study found that different microwave powers could produce different particle sizes and morphologies of final nanocomposite materials, from sheet to rod nanostructures. In chemical syntheses, microwave irradiation can provide thermal and non-thermal effects. Thermal effects of the irradiation process can result in polarization of the molecule without the formation of bonds or the generation of new chemical groups within the molecule itself. This phenomenon increases the material temperature and reaction rate during the synthesis process. In addition, microwave frequencies can stimulate molecules’ vibrations or rotation, resulting in energy level transitions, even to a more active excited state, thereby changing the activation energy (E_a_) and the reaction rate [177]. 

The microwave method is commonly used to intercalate metal/metal oxides into the clay structure because it requires a short time for the synthesis process (less than 15 min). Barakan and Aghazadeh (2016) prepared Al- and Fe-pillared nano-bentonite via microwave irradiation for 3 and 7 min using highly pure aluminum salt (AlCl_3_·6H_2_O) and iron salt (FeCl_3_·6H_2_O) [178]. In their study, the constant molar ratio of Al^3+^:Fe^3+^ was intercalated into the clay structure with a short process at 160 W power irradiation. The results showed that the prepared nanocomposite had a small particle size (less than 1 μm) and a high surface area, almost sixfold higher than the unpillared process. However, this method still has several limitations: the synthesis process can only be carried out on a small/limited scale; a complex process for waste treatment is required; inhomogeneous energy distribution; it is difficult to control the temperature; the flammable organic solvent has limited uses; and the heat generated from irradiation cannot effectively penetrate the container reactor, so in some cases, the synthesis is considerably less efficient [179]. Therefore, Hao et al. (2021) developed the combined microwave–hydrothermal synthesis route for producing Co/CoAl_2_O_4_/sepiolite nanocomposites [180]. The material was prepared under alkaline conditions of 1 mol/L NaOH at 240 °C for 3 h, followed by reduction at 650 °C. The composite materials could easily be obtained by these methods with a high efficiency as a cobalt-based catalyst. The combination and modification of these methods have the advantage that the synthesis can be carried out on large scales.

#### 5.5.3. Ultrasound Irradiation

In recent decades, ultrasound irradiation has been widely used as an alternative energy source in the synthesis of clay-based nanocomposites to achieve green and more sustainable chemical-based synthesis. The effect of ultrasonic waves causes molecules to oscillate because the average distance between the molecules increases, which increases the speed of the synthesis reaction process. Unlike microwaves, ultrasonic irradiation requires lower energy requirements, shorter reaction times, and high product purity [181]. Olaya et al. (2009) prepared pillared clay with Al-Fe-Ce and Al-Fe using ultrasound irradiation in a 30 min reaction process [182]. They found that increasing the concentration of Fe in pillared clay changed the morphology and microporous area of the clay materials. Compared with conventional methods, the developed methods can reduce the consumption of water by up to 90%, as well as reduce the processing time and process cost. The addition of an ultrasonic step to the synthesis process did not change the material properties, such as the catalytic activity and the acidity of the pillared clay. However, the pillarization process with this method produced higher particle size homogeneity than the conventional method. The process of metal/metal oxide pillarization into clay using this method can be optimized by considering several factors, such as ultrasonic irradiation power and viscosity [183]. This method can be used for natural clay pillarization processes by the mixing pillar solution with a clay suspension, using less water and reacting faster. Ultrasound irradiation (20 kHz–10 MHz) from a liquid suspension can cause an acoustic cavitation effect. This process involves generating, growing, and collapsing bubbles in a liquid medium and causes extreme transient conditions, such as pressure in the system in the range of ~500 atm and a high temperature of ~5000 K. Changes in conditions can cause shockwaves induced by cavitation, which trigger the formation of nanostructures [184].

## 6. Clay-Supported Metal Oxide in AOPs

Clay-supported metals and metal oxide nanoparticles could act as catalysts in processes of catalytic oxidation (CO), catalytic wet peroxidation (CWPO), catalytic ozonation, as well as photocatalysts in photocatalytic oxidation (PCPO) and peroxidation (PCO) reactions. Table 3 presents some research on the synthesis and applications of clay-supported metal and metal oxides for AOPs along with the highlighted performances of their activity. 

Catalytic oxidation reactions are based on the catalytic generation of hydroxy radicals in mild conditions, which refers to the high oxidation potential of HO•. More specifically, catalytic wet peroxidation utilizes hydrogen peroxide decomposition by transition metallic cations (M). The catalyst is oxidized by H_2_O_2_, generating HO•, and with additional H_2_O_2_ molecules it will generate hydroperoxyl radicals (HO_2_•), according to the following equations:

M^n+^ + H_2_O_2_ → M^(n+1)+^ + HO• + HO^−^

M^(n+1)+^ + H_2_O_2_ → M^n+^ + HO_2_• + H^+^

M^(n+1)+^ + HO_2_• → M^n+^ + O_2_ + H^+^

In the mechanism discovered by H.J.H. Fenton at the end of the 19th century, highly oxidative properties of hydrogen peroxide were observed in the presence of iron ions during the oxidation of tartaric acid. Thus, the homogeneous Fenton process was developed. The drawbacks of the process are related to the maximum oxidation/degradation efficiency (DE), which could only be achieved in a narrow pH interval (2–4), in addition to non-recoverable metal ions. Forming metals and metal oxides into a stable chemical support demonstrated the feasibility of overcoming these problems. 

Metal and transition metal elements are capable of undergoing this redox reaction due to their oxidation states, which raises the possibility of repetitive oxidation–reduction. A variety of metal and metal oxide catalysts can be utilized as active catalysts, including Fe, Fe_2_O_3_, Fe_3_O_4_, Co, Cu, CuO, ZnO, and ZrO_2_; their immobilization on various solid supports, such as carbon, graphene, zeolite, and clay minerals, has also been reported. Referring to the data in Table 2, the enhanced oxidation rate and degradation efficiency of the CWPO was conclusively obtained by utilizing clay-supported metal and metal oxide. 

The most commonly used metal in Fenton, photo-Fenton, and photo-Fenton-like processes is iron oxide. Its immobilization on clay minerals has been intensively studied for many pollutant compounds consisting of dyes, pesticides, and other persistent organic compounds [102,185,186]. Fe_2_O_3_/montmorillonite prepared in some studies exhibited enhanced catalytic and photocatalytic activity. The increased activity is related to more oxygen vacancies in its pillared form compared with Fe_2_O_3_ nanoparticles (nFe_2_O_3_), which are capable of inducing increased reactive oxygen species (ROS) generation in the presence of a light source. It was reported that the photo-assisted degradation of diethyl phthalate (DEP) was increased by 2.5-fold as compared with nFe_2_O_3_. The surface-bound •OH was the main radical playing a role in the degradation mechanism, and it can be suppressed by the presence of competitor anions such as Cl^−^, NO_3_^−^, and CO_3_^2−^, as well as the pH condition of the solution. The stabilized iron oxide was found to enhance the effectiveness, with insignificant changes in photocatalytic and catalytic activity after three cycles [186]. The homogeneous distribution of the metal oxide on the surface morphology affects the reaction effectiveness, which is also controllable by the dispersion method. As an example, in the synthesis of nano-Fe_2_O_3_/montmorillonite, the impregnation method yielded a different distribution of the nanoparticles as compared with the coprecipitation method [187]. The increasing specific surface area of montmorillonite from 17 to 62 m^2^/g was related to the changed basal spacing (d_001_). 

Minimizing iron leaching during the treatment was another aim of combining Fe with other metals such as Co, Cu, Zn, and Zr. The highly stable catalyst in a wider pH range was attained with Fe/Co-pillared clay with minimal leached iron identified at the end of the reaction: lower than 0.1 mg L^−1^ [148]. In the case of MnO_2_-supported clay, α-MnO_2_ is the dominant phase identified to be inserted into the clay structures, either for montmorillonite or saponite [99,122,188]. The characteristics of high oxidation potential and surface activity of the hydroxyl structure of the phase strongly support the oxidation mechanism. This revealed the rapid and complete oxidation of MB at room temperature without any oxidant [99]. 

Montmorillonite-supported Cu nanoparticles exhibited high degradation efficiency in the CWPO of methylene blue (MB) (95.9%); additionally, clay surface pre-modification with ρ-amino benzoic acid before the nanoparticle support was formed progressively resulted in the complete removal of MB [155]. In this case, immobilizing Cu NPs reduced the surface potential and caused a dramatic fall in the surface area and total pore volume, due to the rupture of the microporous structure. The homogeneously distributed Cu NPs, 8 nm in size in the montmorillonite matrix, represented the effective role of Cu NPs for surface interactions and inducing the oxidation mechanism. Similarly, the supportive adsorption interaction was identified from the CWPO of atrazine using montmorillonite-supported Cu NPs. Mechanistic studies of adsorption and oxidation have revealed the contribution of atrazine adsorption by Cu as an active catalyst via coordination bonding with nitrogen from the atrazine structure, yielding a significant reduction in the chemical oxygen demand of the treated solution. Cu_2_O and CuO identified on the surface are recognized as the redox-active species within the pores, layers, and outside of the montmorillonite surfaces [189]. In an ionic form, Cu^2+^-immobilized aluminum-pillared montmorillonite (Cu/Al-PILM) showed a capability for use in Fenton and photo-Fenton treatments of RO16 [190]. A slit-like mesopore structure with pore diameter of 3.3–3.8 nm and ~6–35 nm was observed in Cu/Al-PILM, with an increasing trend in mesoporosity with the increasingly impregnated Cu^2+^ concentrations. 

The oxide form of CuO nanoparticles not only occupied the interior interlayers, but also the exterior surfaces of Cu/Al-PILM. The mesoporous form significantly contributed to effective heterogeneous catalysis for the removal of RO16. Even though many studies have revealed that clay-supported Cu NPs of CuO expressed stability, the combination of Cu with other metals, such as Fe and Al, in mixed-metal-pillared clay contributed towards resulting in better catalytic properties. The appreciable catalytic activity was reported by the combination of such metal pillaring species with iron, such as Al-Fe-pillared clay or Cu-Fe-pillared clay [128,130]. 

The use of combined metals represented the more effective electron–hole pair oxidation/reduction mechanism. The increasing effectiveness of Zr-immobilized pillared clay in a wastewater model was reported from Zr-Cu/Al-pillared clay for treating wastewater from a winery. The significant improvement in total organic content (TOC) removal from 46% to 60% was exhibited as a synergetic effect of both Zr and Cu on the photocatalytic degradation process. The greater band gap of Zr (5.8–7.1 eV) supported the generation of electron–hole pairs, which migrate to the photocatalyst surface and yield radical species that can react with organic molecules upon redox reactions. The electron transfer process is enhanced by the successive redox of Cu^2+^, a transition metal which continuously releases e-species, induced by the presence of a permanent irradiation source (UV-C). However, starting clay is still the important factor to be considered in the synthesis. The cation exchange capacity, pore structure, and specific surface area strongly determine the capability in the cation exchange process. This considerably affects the final structure of the material as well as the redox activity and stability in the reaction medium [127]. 

Mixed-metal-oxide-pillared clays have been prepared in various compositions and metal sources. One such example is Al/Zr-pillared montmorillonite [191]. An Al:Zr molar ratio of 3:1 was found to be the most active catalyst for the CWPO to phenol. The catalytic activity was strongly influenced by pH, because the zeta potential of the catalyst surface influenced the interaction between the target molecule and the catalyst. The high permanent negative charge on the basal surface of raw clay particles might arise from the high degree of isomorphous substitution of structural Si^4+^ in the tetrahedral layer by polyoxocations from the pillaring agents. This leads to changes in the surface charge arising from the hydroxyl groups at the edges of the parent clay particles. Resulting from the Lewis acidity of the metal precursor, the pillared clays usually have higher zeta potentials than the parent clay [191]. This phenomenon is also reflected in metal-oxide-pillared clay for the PCPO process. Similarly with AOPs utilizing unsupported metal and metal oxides, some influencing parameters, such as pH, oxidant dose, catalyst dose, temperature, target molecule, and reaction time, strongly affect the efficiency of the process. A study on Fe_2_O_3_/montmorillonite for phenol degradation revealed the pH to be the critical factor for influencing the degradation efficiency of phenol removal.

**Figure 13:**
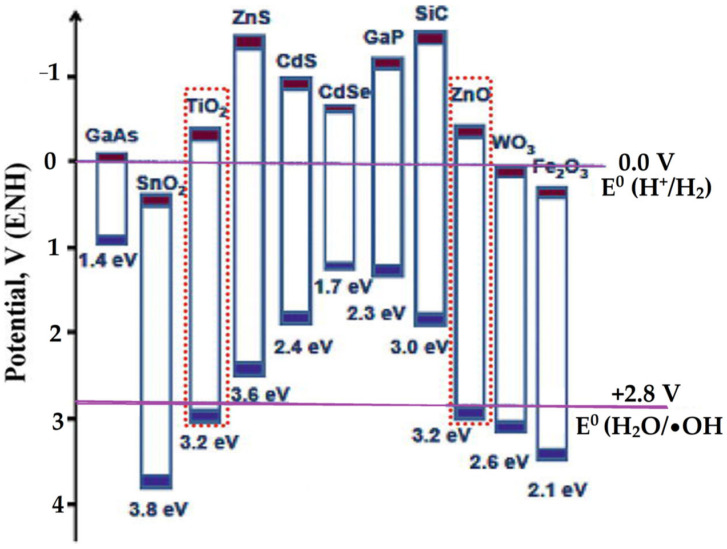
Potential diagram of some semiconductors.

Particularly for photocatalytic oxidation, only the severe metal oxide of semiconductor photocatalysts can be utilized. The basic principle of photocatalysis relies on the generation of electrons and holes as the semiconductor is impinged by photons. In addition, the redox potential of a donor species on the surface of the photocatalyst needs to be more negative (higher in energy) than the valence band position of the semiconductor to fill the electron vacancies. Similarly, acceptor molecules must have a more positive redox potential (lower in energy) than the conduction band. Figure 13 presents the redox potential diagram. 

As an electron is excited from the valence band to the conductance band, •OH radical will be produced from the interaction between H_2_O and the solvent, which then undergoes propagation and complete oxidation of the target molecule in the treated solution. The oxidation mechanism can be summarized by the following equations:

Semiconductor + hv → e^−^ + h^+^_Semiconductor_ + hv → e^−^ + h

h^+^ + H_2_O_ads_ → OH•_ads_ + H^+^h^+^ + H_2_O_ads_ → OH•_ads_

h^+^ + OH^−^_ads_ → OH•_ads_ h^+^ + OH^−^_ads_ → OH•_ads_

e^−^ + O_2_ → O_2_•^−^e^−^ + O_2_ → O_2_•^−^

O_2_•^−^ + H^+^ → HO_2_•O_2_•^−^ + H^+^ → HO_2_•E5

O_2_•^−^ + HO_2_•+ H^+^ → H_2_O_2_ + O_2_O_2_•^−^ + HO_2_• + H^+^ → H_2_O_2_ + O_2_

Enhanced photocatalytic efficiency can be achieved by the higher band gap energy of the semiconductor compared with the redox potential of H_2_O/•OH, as well as the suppressed recombination of electron–hole pairs. The increasing band gap energy, referring to the quantum size effect phenomenon, was reflected by the use of nano-sized metal oxide; moreover, minimizing the recombination can be achieved by trapping the electron or hole in a defect from the conductive support. Sensitization of the metal oxide photocatalyst to overcome the limitations of a narrow-light response range is a strategy for low-cost photocatalysis applicable with sunlight. These strategies were also attempted for clay-supported metal and metal oxide nanoparticles.

TiO_2_ is the most popular photocatalyst, as is its clay-supported metal oxide form. Various kinds of clay minerals, such as bentonite, montmorillonite (Mt), kaolinite, hectorite, laponite, halloysite, palygorskite, and attapulgite, have been utilized as TiO_2_ supports. The use of such Ti precursors as titanium oxide chloride, titanium tetrachloride, titanium isopropoxide, and titanium (IV) butoxides were reported either in the pillarization procedure or impregnation procedure. The particle size of assembled TiO_2_ nanoparticles on the clay support is determined by several factors, including the morphology and clay mineral’s structure. The deposition of TiO_2_ anatase in hectorite, an expandable clay mineral, was found to result in important structural changes at the clay mineral surface for enhancing the photocatalytic activity, compared with its incorporation in kaolinite [192]. In more detail, as a determining factor, the dispersion of TiO_2_ in a nanoflake kaolinite exhibited a smaller TiO_2_ NP size distribution compared with the dispersion in nanorod kaolinite [193]. From various clay-supported-TiO_2_, it can be concluded that a higher adsorptive behavior is the highest contributing performance in photocatalytic activity compared with bulk-TiO_2_, including Degussa P25. From various dye molecule targets in photocatalytic oxidation experiments, the more significant degradation efficiency on cationic dye rather as opposed to anionic dye was identified. In similar reaction conditions of photocatalytic degradation utilizing TiO_2_/montmorillonite, the removal rates were in the order: crystal violet (97.1%) > methylene blue (93.20%) > rhodamine B (79.8%) > methyl orange (36.1%) > Congo red (22.6%) [194]. From the perspective of releasing nanoparticles from the treated solution, montmorillonite plays a role in inhibiting the homo- and hetero-aggregation of TiO_2_ NPs. Depending on the presence of natural organic matter (NOM), pH, and electrolytes, TiO_2_-montmorillonite exhibited higher stability [195]. Some experiments on the sensitization and doping of TiO_2_/clay as a strategy for enhancing photocatalytic efficiency related to the light range and electron hole recombination have been conducted. The use of a ruthenium complex significantly improved the activation capability of TiO_2_/saponite in the visible light region [196]; moreover, the increased band gap energy was achieved by the doping of TiO_2_/montmorillonite and TiO_2_/Kaolinite with Bi [197], W [153], Fe, and N [198,199,200]. W-doped TiO_2_/montmorillonite demonstrated a removal efficiency of atrazine of about 40% under visible light; however, undoped TiO_2_/montmorillonite was almost totally inactive [153]. With the same target molecule of atrazine in a photocatalytic oxidation system, boron-doped TiO_2_/montmorillonite demonstrated a fourfold higher rate than the undoped system. The doped nanocomposite exhibited a favorable phase–junction structure in the visible light region, which could potentially be developed in simpler and less expensive photocatalytic systems [201].

The basic photo-Fenton reaction utilized Fe; however, immobilized Fe and its oxide in clay supports demonstrated excellent photocatalytic activity in the PCPO of various target molecules. Using montmorillonite-supported nanosized zero valent iron (ZVI) via an ion-exchange procedure, followed by mild reduction, produced a homogeneously dispersed ZVI with a mean particle size of 0.86 nm. The stability of the dispersed ZVI was acceptable [154]. Additionally studied were montmorillonite-supported Fe_2_O_3_–Fe_3_O_4_ NPs (NIOM), which have also shown better reusability compared with Fe_2_O_3_–Fe_3_O_4_ NPs. The contribution of the montmorillonite support was critical in decreasing the aggregation and size of the crystals, improving the thermal stability of the crystals. This indicated that the degradation rate of methyl orange when using NIOM was 1.47-fold faster with 45 min of irradiation compared with Fe_2_O_3_–Fe_3_O_4_ NPs [202].

The incorporation of Fe and Fe NPs in metal-oxide-pillared clays is another strategy to enhance the surface activity for producing •OH. The tethering of ferrioxalate in CuO-pillared bentonite has been developed to significantly improve 4-NP photocatalytic oxidation [203]. Ferrioxalate provides an extra source of Fe^2+^ for producing HO^●^, as in the classic Fenton mechanism. The significant effect of the amount of photocatalyst was noticed on the degradation rate.

Zinc oxide nanoparticles are a good alternative to TiO_2_, taking into account its environmentally friendly and low-cost features; therefore, research on the dispersion of ZnO in clay supports has also attracted considerable interest. Zinc oxide has good photochemical, catalytic, and optoelectronic features, which are related to its band gap energy of around 3.4 eV and is included as the wide-band gap II–VI semiconductor. The strong contribution of adsorptive properties of the clay support have triggered many attempts to improve the high supporting capacity by creating Zn in a PCH form. A well-ordered ZnO/clay heterostructure was synthesized using hectorite and saponite by utilizing a sol–gel reaction of the precursors [204,205,206,207]. Similar attempts for photocatalytic activity enhancements were reported through complex attachment and dopants [206].

From the Fe_2_O_3_/montmorillonite and SnO_2_/montmorillonite syntheses, it was revealed that the metal content in the pillaring precursor is an appreciable and crucial factor in determining the surface profile and the particle size of the dispersed metal oxide [123,125]. A higher amount of Sn in the pillaring precursor determined the increasing particle size, which was then correlated with the band gap energy. Furthermore, some factors for AOPs, such as the initial condition of the pollutant, specific surface area, band gap energy, stability, and the presence of oxidants, should be optimized to achieve the best performance, especially in increasing the lifetime of catalysts and photocatalysts. The stability, recyclability, and reusability of the nanocomposite are also important to consider.

**Table 3:** Some clay-supported metal or metal oxide nanomaterial for AOPs applications.

Clay-Supported Metal or Metal Oxide Nanomaterial	Target Molecule	Process	Remark	Reference
MnO_2_ nanosheet/montmorillonite	MB	CO	MB removal achieved 99.89% at 5 min and the catalyst dose of 0.4 g/L	[99]
MnO_2_/montmorillonite	Bisphenol A	CO	Bisphenol A removal was almost 100% after 20 min of treatment	[188]
K-MnO_2_/CeO_2_/Palygorskite	Phenol	CO	90% of phenol removal for the treatment at 130 °C for 103 min	[208]
Fe/Palygorskite	Phenol	CO	CWPO of MB using Cu NPs/montmorillonite gave complete removal	[208]
Fe_2_O_3_/montmorillonite	phenol	PCPO	Complete phenol oxidation reached at 90 min	[185]
Fe_2_O_3_/montmorillonite	Diethyl phthalate	CPO	The material showed stability and reusability with insignificant change of photocatalytic activity until 3 cycles	[186]
Fe_2_O_3_/montmorillonite	Toluene	CO	Complete toluene oxidation reached at 300 °C	[187]
MnO_2_/Al_2_O_3_-pillared montmorillonite	Acetone	CO	Complete acetone oxidation reached at a temperature of 7500 K	[209]
MnO_2_/ZrO_2_-pillared montmorillonite	Acetone	CO	40% of acetone oxidation reached at a temperature of 7500 K	[209]
Cu NPs/montmorillonite	Methylene blue (MB)	CWPO	CWPO of MB using Cu NPs/montmorillonite gave complete removal	[155]
Cu NPs/montmorillonite	Atrazine	CWPO	Nanomaterials exhibited adsorption and catalytic oxidation activity for atrazine removal with DE of 82.12% and 85.94%, respectively	[189]
Cu-impregnated Al-pillared montmorillonite	Reactive orange 16 (RO16)	CWPO PCPO	Complete removal of RO16 after 90 min by both AOP mechanisms	[190]
Fe/Co-pillared clay	Paracetamol	CWPO	Optimum condition for completely paracetamol removal was treatment for 6 h, H_2_O_2_ concentration of 472 mg L^−1^, catalyst dose of 2.5 g L^−1^, temperature of 80 °C, and initial pH = 3.5	[190]
Zr-pillared clay	4-nitrophenol	CWPO	Complete removal at 4 h with small amount of H_2_O_2_ and catalyst loading of 2.5 g/L)	[210]
Al/Zr-pillared clay	Phenol	CWPO	The optimum condition for the CWAO process is a pH of 3, reaction temperature of 100 °C, catalyst dosage of 2 g/L, and oxygen pressure of 10 bar. The reaction obeys the first-order power rate law kinetics model with the apparent activation energy of 21.306 kJ/mol	[191]
Zr immobilized in Cu/Al-pillared clay	Winery wastewater	CWPO	The presence of Zr enhanced the oxidation capability of the catalyst	
Fe- and Cu-immobilized in Zr-pillared clay (Fe/Cu/Zr-APILC)	4-nitrophenol	CWPO	Complete removal after 2 h; the highest TOC removal (65.1% after 8 h) was obtained with Fe/Cu/Zr-APILC	[210]
Co-immobilized AL-pillared clay	Tartrazine	CO	Co^2+^ was impregnated onto aluminum-pillared clay and utilized as tartrazine oxidation via PMS	
Copper-pillared ferrioxalate-modified bentonite (Cu/PBC)	4-nitrophenol	PCPO	Maximum DE of 99.89% was achieved with an excess of H_2_O_2_, and catalyst loading of 2.0 g/L during 6 min of visible light illumination.	[203]
Al–Fe-pillared clay	4-NP	CWPO	Maximum DE of 99.7% with TOC removal and COD removal of 83.6% and 75%, respectively, attained after 300 min with an excess of H_2_O_2_ at 50 °C	[130]
Al–Cu–Fe-pillared clay	4-NP	CWPO	Maximum DE of 99.7% with TOC removal and COD removal of 63% and 65%, respectively, attained after 300 min with an excess of H_2_O_2_ at 50 °C	[130]
Al–Cu PILCs	4-NP	CWPO	Maximum DE of 99.7% with TOC removal and COD removal of 60% and 55%, respectively, attained after 300 min with an excess of H_2_O_2_ at 50 °C	[130]
Al/Fe-, and Al/(Fe–Cu)- bentonite	Methyl orange (MO)	CWPO	The Al/Fe-pillared bentonite attained the complete removal of MO after 1 h of reaction at room temperature	[128]
Cu-doped Fe-pillared Tunisian clay (Cu/Fe–PILC)	Phenol	PCPO	Cu/Fe–PILC demonstrated stability for a wide range of pH, from 3 to 7, for the PCPO process of phenol removal. Nanocomposite showed reusability with negligible metal leaching without a noticeable loss of activity	[211]
Fe-pillared clay (Fe-PILC)	Phenol	PCPO	Phenol removal efficiency of 100% was achieved after 60 min of photocatalytic oxidation reaction UV 254 nm	[86]
Fe_2_O_3_-Fe_3_O_4_ nanoparticles (NIO) supported in montmorillonite (NIOM)	MO	PCPO	NIOM exhibited a higher photocatalytic activity compared with Fe_2_O_3_–Fe_3_O_4_	[202]
Fe_2_O_3_/kaolin	Rhodamine B (RhB)	PCPO	DE of 98% by using 1 g/L of catalyst and 0.05 mol/L of H_2_O_2_ for 120 min. The Fe_2_O_3_–kaolin catalyst displayed high photocatalytic activity in a wide pH range of 2.21–10.13	

## 7. Conclusions and Future Perspective

AOPs are promising methods and advanced treatment processes for the degradation and mineralization of various pollutants, either in air or in water; particularly, AOPs have attracted considerable interest for wastewater treatment. The most extensively studied AOPs are catalytic oxidation, catalytic wet peroxidation, photocatalysis, and photooxidation, which have demonstrated their efficiency in the removal of recalcitrant compounds from pollutants. The immobilization of semiconductors or metal/metal oxide nanoparticles on clay structures is one such effort to support and enhance the effectiveness of the catalytic process. Although the uses of clay-supported metal/metal oxide nanoparticles in AOPs are highly successful at laboratory scales, there are not many successful examples of their applicability at industrial scales. The economic viability and efficiency of AOPs are key factors that limit their industrial applications. Such pilot schemes for nanocomposite setups at larger scales require considerable optimization and face some adaptations from the laboratory scale. In addition, the synthesis of clay-based nanocomposite is usually evaluated in relation to the long synthesis process and its reproducibility in larger amounts. Minimizing water intensification for the synthesis is the aspect that requires the most consideration. However, compared with some other inorganic supports for metal/metal oxides, such as mesoporous silica, synthetic zeolite, alumina, carbon, and graphene, the use of clay minerals in this study summarizes the existing higher potential for the AOP treatments, from perspectives of cost and resource use. Future studies should focus on the intensification of scaling-up the applications of clay-supported metal/metal oxide to develop effective and low-cost AOPs. This will be useful to support small- and medium-scale industries that release pollutants.

## Figures and Tables

**Table 1:** Metal oxide nanoparticles for AOP applications.

Metal/Metal Oxide	Precursor and Synthesis Method	Remark	Reference
Mn_3_O_4_	Precursor of MnCl_2_, by the precipitation method	Particle sizes of Mn_3_O_4_ nanoparticles are 20–35 nm, Mn_3_O_4_ NPs show high activity for the 99.9% degradation of methylene blue by photooxidation	[48]
ZnO	Zinc acetate precipitation method	ZnO NPs show a removal efficiency of reactive blue of 85.4%	[49]
γ-Fe_2_O_3_	Synthesized using FeSO_4_·7H_2_O by the precipitation method	γ-Fe_2_O_3_ NP sizes of 40–50 nm with a phenol degradation activity of 94.5% within 420 min at 80 °C. The NPs shows reusability until the fifth cycle	[50]
α-Fe_2_O_3_	α-Fe_2_O_3_ NPs were in nanocubic form, prepared using a metal-ion-mediated hydrothermal route.	α-Fe_2_O_3_ NPs showed photocatalytic activity in rhodamine B degradation	[51]
CuO	CuO NPs were prepared by reflux and precipitation methods, followed by calcination at different temperatures of 350–550 °C. Particle’s sizes are in the range of 17–34 nm, depending on method and calcination temperature	The highest activity of CuO NPs was exhibited by the NPs prepared by the reflux method and calcined at 450 °C. The highest degradation efficiency toward phenol was 95%. Nanoparticles showed stability until the third cycle	[52]
SnO_2_	SnO_2_ in nanosphere form was synthesized through a solvothermal method by a SnCl_2_ precursor	The highest degradation efficiency of rhodamine B was 99% during 90 min under UV light	[53]

**Table 2:** Some pillared clays syntheses and highlighted crucial factors for the syntheses.

Pillared Clay	Precursor	d_001_ (nm)	Crucial Factor	Ref
Fe_2_O_3_-rectorite	Na_2_CO_3_ solution in mixture with Fe(NO_3_)_3_ at the molar ratio Na:Fe of 1:1, under stirring at 25 °C	0.25	-	[79]
TiO_2_/montmorillonite	Tetrabutyl titanate in mixture with HCl by slowly dropping 0.5 of [Ti]:[H^+^] molar ratio under stirring 1.0 h	0.357	Calcination temperature determined the specific surface area	[113]
TiO_2_/montmorillonite	Various pillaring precursor: Ti-PILCs consist of adding TiCl_4_ to a solution of HCl, which can be diluted with deionized water under vigorous stirring to obtain hydrolyzed Ti^4+^	0.2–0.3	HCl:Ti molar ratio; temperature at which the pillaring solution is prepared; clay suspension concentration; mmol of Ti:clay ratio; and calcination temperature	[122]
Fe_2_O_3_-pillared bentonite	FeCl_3_ and NaOH at a molar ratio ^−^OH:Fe = 1:1, stirred at room temperature overnight	0.06–0.84	Fe molar-to-mass ratio influencing the specific surface area and basal spacing d_001_	[123]
Fe_2_O_3_-pillared montmorillonite	Trinuclear acetate Fe(III) ion, [Fe_3_(OCOCH_3_)_7_OH·2H_2_O]	0.1	Fe molar-to-mass ratio influencing the specific surface area	[111]
Fe_2_O_3_-pillared montmorillonite	FeCl_3_ and NaOH at the molar ratio ^−^OH:Fe = 1:1, stirring for 4 h at room temperature	-	^−^OH:Fe molar ratio, Fe content and calcination temperature are the important parameters influencing the character of pillared clay	[124]
SnO_2_/montmorillonite	Slowly titrated with NaOH and SnCl_2_ solution with Sn:OH molar ratio of 1:1, stirred overnight	1.4–1.6 nm	Sn molar-to-mass ratio influencing the specific surface area, basal spacing d001, and particle size of SnO_2_	[125]
Cu/Al-pillared bentonite	Cu^2+^/(Al^3+^+Cu^2+^) molar ratios 0, 0.05, 0.1, 0.15 and 0.2.	0.7–0.88	Cu^2+^/(Al^3+^+Cu^2+^) molar ratios determined the increasing d_001_ and specific surface area	[119]
TiO_2_-pillared montmorillonite	The mixture of HCl-Ti isopropoxide at the HCl:Ti molar ratio of 10		The calcination by microwave irradiation influenced by the power of microwave	[126]
Al/Fe-pillared clay	AlCl_3_ in mixture with FeCl_3_ titrated with NaOH		The starting clay	[127]
Al/Fe-pillared clay	AlCl_3_ in mixture with FeCl_3_ titrated with NaOH	0.45	Al:Fe molar ratio influenced the physicochemical character of material	[128]
SnO_2_/montmorillonite	SnCl_2_ in mixture with NaOH at the Sn:OH molar ratio of 1:1		Sn:montmorillonite mass ratio influenced the character of materials	[80]
TiO_2_-pillared montmorillonite	Titanium isopropoxide-HCl	0.25	Microwave power influenced the physicochemical character of material	[129]
Al-Fe pillared clay	FeCl_3_ and AlCl_3_ with the molar ratio of Al:Fe = 5:1, titrated with NaOH under vigorous stirring to obtain a molar ratio of OH:(Al+Fe) = 2	0.46	Al:Fe and OH:(Al+Fe) = 2 determining the basal spacing d_001_ and specific surface area	[130]
ZnO/sepiolite heterostructure	Zn-acetate and KOH in methanol under precipitation method	Not reported	Material has capability to be support for Fe_2_O_3_	[131]
SnO_2_/bentonite	SnCl_2_ at various contents (10, 20, and 30 wt.%) in mixture with NaOH at the pH of 11–12, stirred at 60 °C	Not reported	Material has the capability to effectively degrade MB	[132]
TiO_2_/sepiolite	Tetrabutyl titanate (TBT) and acetic acid under solvothermal	Not reported	Material has the capability to effectively degrade MB	[133]
TiO_2_/montmorillonite	Titanium tetraisopropoxide was added to a vigorously stirred acetic acid solution of 80 wt.%. The resulting white slurry was stirred at 323 K to give a clear TiO_2_ sol	0.48	Kind of clay determined the hydrophobicity of pillared clay	[134]
TiO_2_/montmorillonite	TiCl_4_ was diluted with CH_2_Cl_2_ to obtain a clear solution. Then, the mixture was slowly added to Na–M suspension under vigorous stirring at 65 °C for 4 h under reflex system	1.60	The Ti content in TiO_2_–M was 48.6 wt.% with an anatase crystallite size of about 15–20 nm	[135]
ZrO_2_/bentonite	A zirconium polycation solution was prepared by the slow titration of a ZrCl_4_ solution (0.1 M) with a solution of NaOH (0.2 M) under vigorous stirring, using an OH:Zr molar ratio equal to 4:1.	0.95	Ageing temperature of intercalated bentonite influences the distribution of polyoxocations	[136]
TiO_2_/montmorillonite	Titanium polycation solution was prepared by the hydrolysis of TiCl_4_	0.20	Hydrothermal treatment and calcination temperature influenced the increasing d_001_ and titanium dioxide phase	[118]

## Data Availability

This study did not report any data.
